# *Borrelia burgdorferi* Keeps Moving and Carries on: A Review of Borrelial Dissemination and Invasion

**DOI:** 10.3389/fimmu.2017.00114

**Published:** 2017-02-21

**Authors:** Jenny A. Hyde

**Affiliations:** ^1^Department of Microbial Pathogenesis and Immunology, College of Medicine, Texas A&M Health Science Center, Bryan, TX, USA

**Keywords:** *Borrelia burgdorferi*, Lyme disease, dissemination, invasion, vascular interaction, protease, motility, chemotaxis

## Abstract

*Borrelia burgdorferi* is the etiological agent of Lyme disease, a multisystemic, multistage, inflammatory infection resulting in patients experiencing cardiac, neurological, and arthritic complications when not treated with antibiotics shortly after exposure. The spirochetal bacterium transmits through the *Ixodes* vector colonizing the dermis of a mammalian host prior to hematogenous dissemination and invasion of distal tissues all the while combating the immune response as it traverses through its pathogenic lifecycle. The innate immune response controls the borrelial burden in the dermis, but is unable to clear the infection and thereby prevent progression of disease. Dissemination in the mammalian host requires temporal regulation of virulence determinants to allow for vascular interactions, invasion, and colonization of distal tissues. Virulence determinants and/or adhesins are highly heterogenetic among environmental *B. burgdorferi* strains with particular genotypes being associated with the ability to disseminate to specific tissues and the severity of disease, but fail to generate cross-protective immunity between borrelial strains. The unique motility of *B. burgdorferi* rendered by the endoflagella serves a vital function for dissemination and protection from immune recognition. Progress has been made toward understanding the chemotactic regulation coordinating the activity of the two polar localized flagellar motors and their role in borrelial virulence, but this regulation is not yet fully understood. Distinct states of motility allow for dynamic interactions between several *B. burgdorferi* adhesins and host targets that play roles in transendothelial migration. Transmigration across endothelial and blood–brain barriers allows for the invasion of tissues and elicits localized immune responses. The invasive nature of *B. burgdorferi* is lacking in proactive mechanisms to modulate disease, such as secretion systems and toxins, but recent work has shown degradation of host extracellular matrices by *B. burgdorferi* contributes to the invasive capabilities of the pathogen. Additionally, *B. burgdorferi* may use invasion of eukaryotic cells for immune evasion and protection against environmental stresses. This review provides an overview of *B. burgdorferi* mechanisms for dissemination and invasion in the mammalian host, which are essential for pathogenesis and the development of persistent infection.

## Introduction

Lyme disease, the leading tick-borne infection in the United States, occurs in multiple stages and is a multisystemic disease due to the etiologic agent *Borrelia burgdorferi* (1–4). This disease was first recognized during the 1970s when a cluster of rheumatoid-like arthritis cases occurred in patients who were predominantly children in Lyme, Connecticut (5). Lyme disease has become a significant emerging infectious disease with the Centers for Disease Control (CDC) estimating approximately 300,000 new cases in 2013 in the United States (6). The spirochetal bacterium is transmitted when an *Ixodes* tick vector colonized with *B. burgdorferi* takes a blood meal on reservoir mammals, such as small rodents and birds, or accidental human hosts, resulting in the colonization of dermal tissue and develops into a localized infection (7). This earliest stage of Lyme disease is characterized by a painless bulls-eye rash, called an erythema migrans, experienced by approximately 70–80% of patients at the site of the tick bite (1–3). In the absence of the distinctive erythema migrans, Lyme disease can be difficult to diagnose due to non-specific flu-like symptoms including headache, neck stiffness, malaise, fatigue, myalgia, and fever. During localized infection, the number of *B. burgdorferi* cells increases in the dermal tissue in preparation for dissemination to sites of secondary colonization. Days to weeks after infection, *B. burgdorferi* progresses onto a disseminated stage when the pathogen travels away from the site of the tick bite through the bloodstream and/or lymphatic system to invade and colonize various tissues, such as the heart, synovial fluid of joints, and the nervous system. Patients can develop secondary erythema migrans at distal locations on the skin from the original site of infection. Late infection, the third stage of disease, develops months to years after exposure to *B. burgdorferi* and patients can experience different manifestations including neuroborreliosis, Lyme carditis, and/or arthritis. Following antibiotic treatment, a subset of patients continues to present with arthritic symptoms that has been designated postinfectious, antibiotic-refractory Lyme arthritis (8).

*Borrelia burgdorferi* is a gram-negative “like” bacterium that is a member of the Spirochaetaceae family known by its distinctive spiral morphology and motility propelled by the endoflagellum (9, 10). Motility of *B. burgdorferi* is essential for pathogenesis throughout the enzootic cycle, though swimming traits differ greatly in the *Ixodes* tick than that observed in the mammalian host (11, 12). The segmented genome of *B. burgdorferi* is unlike most bacterial pathogens consisting of a 910,725 bp linear chromosome along with up to 23 circular and linear plasmids ranging in size from 5 to 56 kb (13, 14). The borrelial genome is minimal in size relative to other bacterial pathogens but is also limited in genes annotated to encode metabolic capabilities, virulence, and defense mechanisms. The glycolytic pathway is the only complete metabolic pathway identified in the annotated genome while pathways for generating energy, amino acids, and lipid synthesis, to name a few, are noticeably incomplete or absent (15, 16). *B. burgdorferi* scavenges from its environment to fulfill basic needs for survival and the pathogen goes as far as incorporating host lipids into the outer membrane (OM) that could also serve as an evasion mechanism (17). The simple bilayer membrane structure and limited number of detoxification genes provides little protection to *B. burgdorferi* from the onslaught of the host immune response, specifically oxidative and nitrosative stress (17–22). Borrelial oxidative stress regulator (BosR) has been associated with regulating genes involved in combating oxidative stress and is important for establishing mammalian infection (23–31). A single superoxide dismutase (sodA) aids in the resolution of reactive oxygen species (ROS), but to date a functional catalase has not been identified (32, 33). The reduced form of coenzyme A (CoASH) has been shown to serve in the place of catalase in *B. burgdorferi* by reducing hydrogen peroxide (22). Dynamic temporal and spatial regulation of borrelial genes is crucial for successful colonization, dissemination, and invasion of *B. burgdorferi* in the tick vector and mammalian host (7, 34, 35). Borrelial two-component pathways HK1/Rrp1 and Rrp2-RpoN-RpoS in the tick vector and mammalian host, respectively, regulate genes with a variety of functions including metabolism, chemotaxis, antigenic variation, and adhesion (7, 34). *B. burgdorferi* gene regulation also contributes to immune evasion as it progresses through the different stages of murine infection by inducing the recombination events of an antigenic variation gene (vlsE) and the activation or repression of lipoprotein genes in a tissue specific and temporal manner (36). The lack of secretion systems or toxins limits the ability of *B. burgdorferi* to modulate the resulting inflammatory disease.

Escape of *B. burgdorferi* from the tick midgut to the hemolymph during a blood meal is an important step for transmission through the salivary glands to a mammalian host providing the necessary environmental cues, such as temperature and pH, for adaptation (7, 34). *B. burgdorferi* gene regulation in response to environmental cues is the best characterized for the early localized stage of infection, but much remains to be understood about the mechanisms for dissemination and invasion necessary for the later stages of Lyme disease (7, 35, 37). Dissemination is a necessary step in pathogenesis, and this process inhibits clearance by the immune system, but the specific mechanisms and host–pathogen events that initiate and complete this stage of infection are not well understood. Invasion of *B. burgdorferi* allows the pathogen to reach immunoprotected niches in the mammalian host where the pathogen is not cleared, but induces inflammation. The intimate interaction between *B. burgdorferi* lipoproteins and vascular tissue, in particular with extracellular matrices (ECM), has been shown to be integral to borrelial pathogenesis (38–41). *B. burgdorferi* adhesin interactions with the ECM and/or requirements for mammalian infection have been thoroughly reviewed by several contributors in the field (38–40), but the specific roles for many of these adhesins in dissemination and/or invasion has not been evaluated, therefore, were not addressed in this review. The purpose of this review is to highlight the mechanisms utilized by *B. burgdorferi* for dissemination and invasion of tissues of human and reservoir host.

## Morphology, Motility, and Chemotaxis Regulation

A discussion of dissemination and invasion would be incomplete without briefly describing the unique morphology, motility, and chemotaxis of *B. burgdorferi* that has been previously thoroughly reviewed (10, 12, 42). *B. burgdorferi* is often referred to as a gram-negative “like” bacterium because it is void of lipopolysaccharide (LPS) in its OM, but has a similar membrane organization. The unique helical shape of spirochetes is due to its composition of the lipid bilayer OM, a periplasmic space containing peptidoglycan and endoflagella, and a second lipid bilayer inner membrane (IM) forming the inner most compartment of the cell structure (also called the protoplasmic cell cylinder). *B. burgdorferi* cells are long in length and thin in diameter with dimensions of 10–20 µm and approximately 0.3 µm, respectively. We have limited knowledge about the role of peptidoglycan in the morphology and motility of *B. burgdorferi*, but recent work has demonstrated that the temporal and spatial regulation of peptidoglycan synthesis occurs separately from cell elongation and septum formation take place separately, thus potentially contributes to the unique cell shape (43). In the periplasmic space, a flagellum is attached to each pole then travels toward the midline of the cell and potentially overlaps with the flagellum anchored at the opposite end of the cell (10, 12). Multiple flagellum, ranging from 7 to 11 individual flagellum, cluster at the poles of the cell and arrange themselves into a ribbon to form the borrelial endoflagella that wraps around the periplasmic cylinder generating the characteristic helical or planar flat-wave morphology. The concealment of the flagella within the periplasmic space preventing recognition by the host innate immune response is a passive means of immune evasion by *B. burgdorferi*.

**Figure 1:**
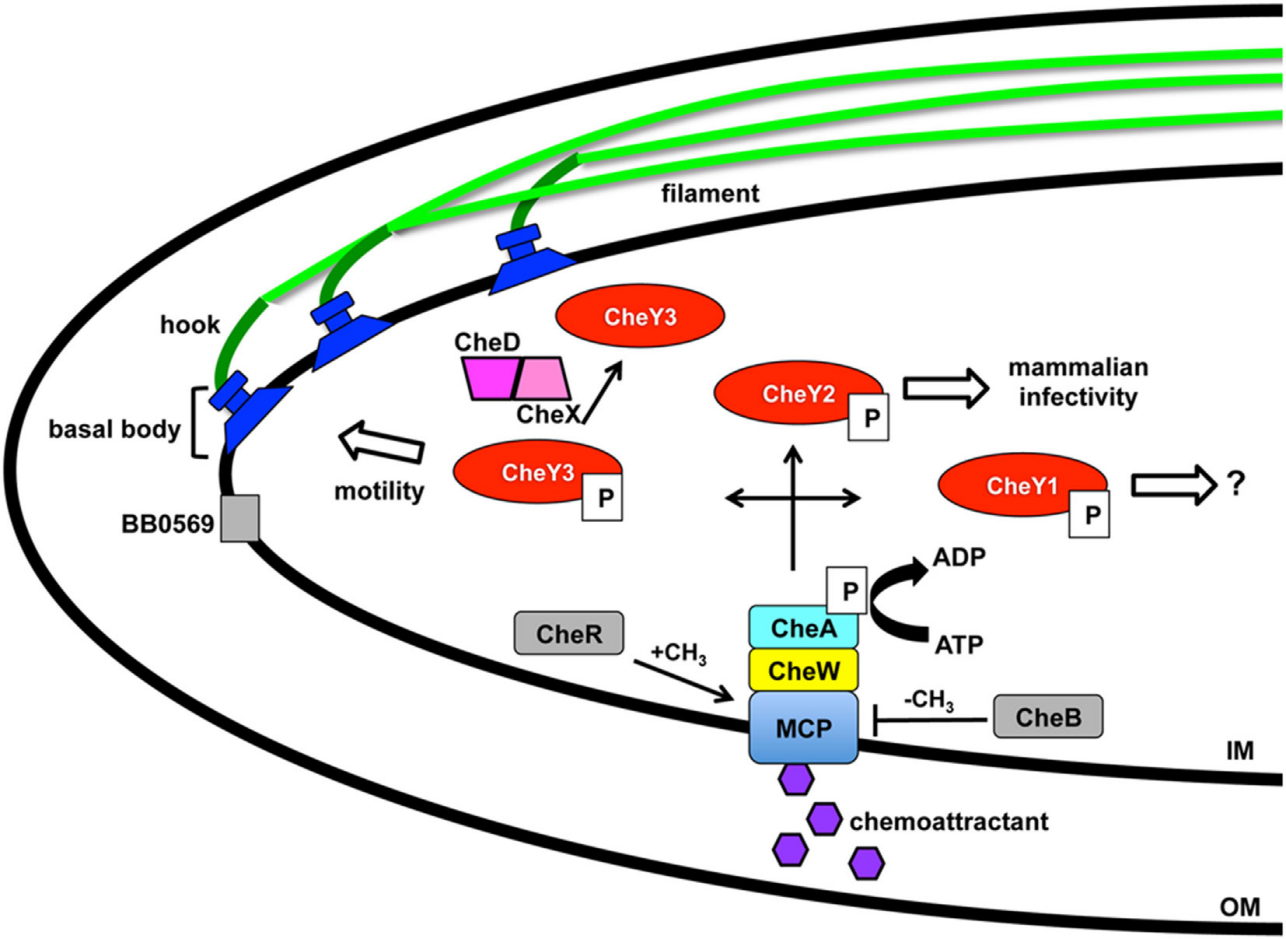
***Borrelia burgdorferi* motility and associated regulation**. Motility of *B. burgdorferi* is driven by multiple flagellum in the periplasmic space that are comprised of an inner membrane anchored basal body, hook, and filament. Coordinated rotation of the flagellar motors drive the direction of swimming or causes flexing of the spirochete. A two component regulatory system controls the motility in response to chemoattractant signals through the membrane-bound chemoreceptor and methyl-accepting chemotaxis protein (MCP). A complex is formed between MCP, linker protein CheW, and histidine kinase CheA with the latter able to autophosphorylate and transfer the phosphate to response regulators CheY1, CheY2, and CheY3 resulting in distinct outcomes. Levels of CheY-P can be reduced by the phosphatase CheX that forms a complex with CheD and increases its activity. The borrelial proteins designated in gray are chemotaxis homologs that have not been characterized to date. BB0569 localizes to the poles of the cell near flagellar motors and influences motility, but its specific function is unknown. CheR and CheB are potential methyltransferase and methylesterase proteins, respectively, which may modify MCP. Modification of Xu et al. (54).

The intricacy of the borrelial cell structure and complexity of its motility program makes the accompanying regulation astoundingly complex. Motility of each flagella in the cluster requires the coordinated activities of the basal body, hook, and filament (10, 12) (Figure 1). The basal body includes the export apparatus (FliH and FliI), C-ring switch complex (FliG and FliM), MS ring (FliF), collar structure, FliL, rod (FliE), P-ring, and the motor stator (MotA and MotB). The export apparatus and C-ring are located on the cytoplasmic face of the IM and associates with the MS ring that is flanked by FliL and stator embedded in the IM. FliL is important for the orientation of the motor and localizes between the stator and rotor. MotB, a motor protein, is required for *in vivo* helical morphology and motility (44). The rod extends from the MS ring into the P ring prior to transitioning into the flagellar hook. The rod and P-ring flank the collar and genes involved in collar formation have not been identified in *B. burgdorferi*. Recently identified, FlbB is a novel flagellar motor protein that associates with the basal body to achieve the proper orientation of the flagella and collar assembly (45). The primary contributor to the flagellar hook is FlgE that connects to the filament composed of FlaA and FlaB that are exported by a SecA-mediated pathway and Type III flagellin-specific pathway, respectively (10, 12). Coordinated motility of polarized endoflagella is a complex process for directional swimming or non-directional flex modality of borrelial cells. Endoflagella anchored on opposite ends rotation is coordinated to drive swimming in a single direction by one flagella moving clockwise and the second counterclockwise. Reversal of swimming occurs when both endoflagella change direction of rotation. The non-directional mode of motility occurs when the both endoflagella move in a clockwise or counterclockwise rotation resulting in a flex state that is similar to the tumble pattern of movement observed for other bacteria (46). *B. burgdorferi* motility in conventional liquid cultivation is widely homogeneous with the cells in a constant swim state. However, the *in vivo* environments in which the pathogen traverses throughout the enzootic cycle are rarely consistent. *In vitro* cultivated *B. burgdorferi* in a media complex with 3% gelatin displayed heterogeneous motility patterns not unlike that observed in mammalian dermis (47). Another morphology taken on by *B. burgdorferi* is a round body, also referred to as a cystic formation, which is a proposed reversible survival mode when under conditions of stress (48–53). The mechanism for round body formation and the role of borrelial endoflagella in this process is unknown.

Borrelial motility is required for virulence, but more specifically is essential for the invasion of tissues in response to environmental signals (10, 12). A two-component signal transduction pathway regulates *B. burgdorferi* chemotaxis and utilizes a membrane-bound chemoreceptor and methyl-accepting chemotaxis protein (MCP) that forms a complex with linker protein CheW and histidine kinase CheA to sense environmental cues (Figure 1). CheA autophosphorylates prior to transferring the phosphate to three response regulators, CheY1, CheY2, and CheY3, encoded by *B. burgdorferi*. A low concentration of CheY-P initiates a run motility and high CheY-P concentrations causes *B. burgdorferi* to flex. CheX phosphorylase rapidly reduces the amount of CheY-P and is enhanced by forming a complex with CheD (55, 56). CheY3 is the only CheY response regulator that has a role in motility and CheY1 or CheY2 are not able to complement the function of CheY3 (57). Phosphorylated CheY3 interacts with the flagellar switch proteins to regulate the rotation of the motors. Xu et al. recently demonstrated a requirement of CheY2 for infectivity. However, this protein does not play a role in chemotaxis or motility (54). *B. burgdorferi* encodes homologs for chemotaxis adaptation proteins CheB and CheR, but to date, these proteins have not been characterized. In *E. coli*, CheR and CheB alter the methylation of MCP by methyltransferase and methylesterase activity, respectively, in response to positive and negative stimuli (46). The number of chemotaxis homologs encoded in the *B. burgdorferi* genome is greater and far more complex than other motile pathogens, such as *E. coli* or *S. enterica* (13, 14). Additional genes, *csrA* and *bb0569*, influence the motility regulation of *B. burgdorferi* (58, 59). Carbon storage regulator, CsrA, is involved in posttranscriptional regulation of flagellar genes and contributes to cell morphology (59, 60). BB0569, annotated as a hypothetical protein, has sequence similarity to MCPs and was found to localize to the poles of the borrelial cell and important for motility and chemotaxis (58). *B. burgdorferi* motility is regulated throughout the enzootic cycle with the distinct behavior of intermittent swimming in the tick vector that includes periods of a non-motile state relative to the persistent swimming observed in the murine host (44, 61).

## Dissemination

*Borrelia burgdorferi* disseminates from the site of the tick bite within the mammalian host to secondary colonization sites requiring dramatic adaptation of the pathogen in response to environmental changes and obstacles presented by the host innate immune response (7, 62–65). A bottleneck occurs during the *B. burgdorferi* initial colonization of dermal tissue when establishing localized infection. This process is in part mediated through the MyD88 pathway, which potentially alters the spirochetal population progressing into the dissemination stage when it infects distal tissues (66–70). *B. burgdorferi* dissemination in mammals occurs by hematogenous and non-hematogenous routes, such as through the lymphatic system or direct spread through the tissues, with the former being the most well understood (71, 72). *B. burgdorferi* hematogenous dissemination has been the focus of much research identifying distinct infectivity classifications of environmental or human isolates by the ribosomal spacer type (RST) or ospC sequence heterogeneity (73). Three RST genotypes have been defined for *B. burgdorferi* as RST1, RST2, and RST3 in descending order of dissemination capabilities (71, 74). At least 16 different *ospC* genotypes that vary in the ability to disseminate and cause disease have been recognized (73, 75–80). More human infections have been associated with *ospC* types A, B, I, H, and K, but there is no correlation with the frequency distribution of *ospC* genotypes in infected *Ixodes scapularis* (73, 81). Genotypes A and B are part of RST1, type I is in RST2 and H and K in RST3 (73). Genotypic differences can predict the ability of a *B. burgdorferi* strain to disseminate and cause infection. The effectiveness or lack of physical barriers, host factors, and immune response can also influence the outcome of borrelial infection.

### *B. burgdorferi* Hematogenous Vascular Interactions

After the establishment of a localized dermal infection, *B. burgdorferi* begins to disseminate throughout the mammalian host *via* the hematogenous route (3, 71, 82). *Borrelia hermsii*, etiologic agent of relapsing fever, reaches high bacterial loads in the blood. However, *B. burgdorferi*, which also disseminates by hematogenous route, is difficult to isolate from blood due to the low number of bacterial cells present. The monitoring of bioluminescent *B. burgdorferi* in real time during murine experimental infection with an *in vivo* imaging system (IVIS) demonstrated the pathogen established a strong localized infection signified by elevated emission of light centered around the site of inoculation followed by dissemination throughout the skin (83). The inoculum dose altered the spatial and temporal dissemination of *B. burgdorferi* constitutively expressing codon-optimized firefly luciferase, but the general pattern of dissemination was the same independent of dose (83, 84). Upon reaching specific tissues or target sites, *B. burgdorferi* traverses dense extracellular matrix (ECM) and crosses tissue barriers through yet to be elucidated mechanisms.

**Figure 2:**
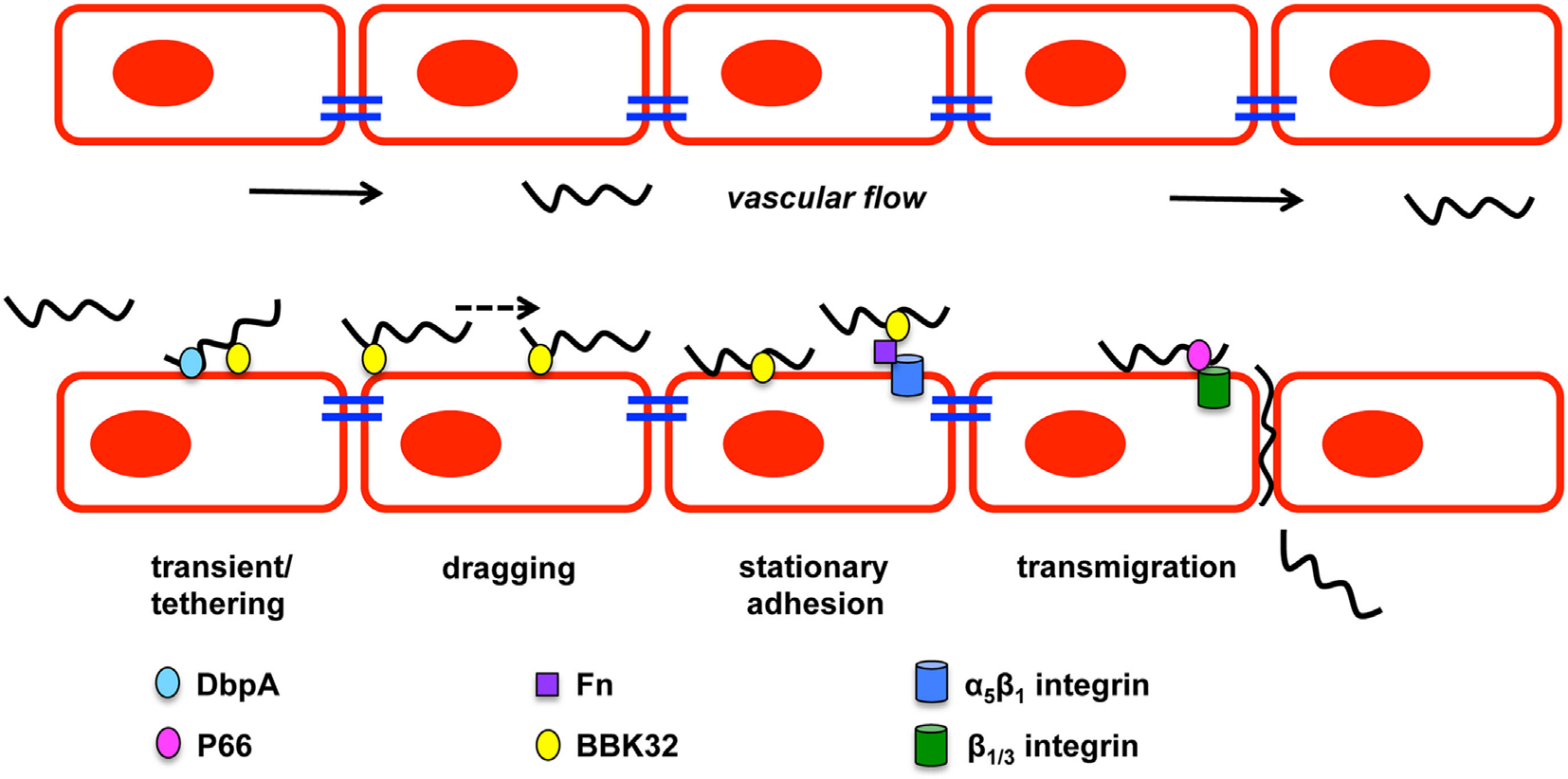
***Borrelia burgdorferi* vascular interactions and transmigration**. *B. burgdorferi* forms three types of vascular interactions: transient/tethering, dragging, and stationary adhesion. Decorin-binding protein A (DbpA) promotes transient/tethering with endothelial cells. Fibronectin-binding protein (BBK32) is involved in all three types of interactions. Stationary interaction through BBK32 may be through direct interaction with the endothelial surface or through a fibronectin bridge (Fn) to a host integrin. P66 also interacts with integrins and is important for transmigration of *B. burgdorferi*. Figure is a modification of Moriarty et al. (86).

Hematogenous dissemination of pathogens within mammals is poorly understood due to the difficulty of evaluating bacteria under the shear stress conditions in the blood stream. Shear stress is a type of tangential stress that acts along a parallel surface due to friction caused by fluid viscosity that is overcome by bacteria for dissemination and invasion (85). The utilization of intravital microscopy (IVM) and spinning disk confocal intravital IVM allowed the evaluation of biofluorescent *B. burgdorferi* in real time under *in vivo* shear stress conditions that characterized the multiple stages of vascular interaction prior to transmigration (86–88). GFP-expressing *B. burgdorferi* visualized by IVM in murine ears approximately 3–4 weeks after infection demonstrated swimming and flexing motility commonly observed under *in vitro* cultivation (86). Microvascular interactions visualized in the murine skin flank by epifluorescence and spinning disk confocal IVM following intravenous injection of biofluorescent *B. burgdorferi* found that bacterial cells did not localize to arterioles, but localized to capillaries, postcapillary venules, and large veins. The study defined *B. burgdorferi* dissemination interactions as transient tethering-type associations, dragging interactions, and stationary adhesion (Figure 2). It is important to note that not all of the *B. burgdorferi* cells associated with the vasculature and thus were not directly assessed (86). Rapid extravasation of *B. burgdorferi* that did not form prior interactions was observed from postcapillary venules. Transient tethering-type interactions occurred briefly when *B. burgdorferi* slowed down relative to the velocity of the blood stream to associate with the endothelium at one region or tip of the cell in a manner that appeared to tether the cell to the surface followed by a detachment. Tethering stabilizes interactions by reducing the dissociation rates of *B. burgdorferi* from the endothelium (89). Dragging, another type of short-term interaction, was distinguished from tethering by *B. burgdorferi* coming into contact with the endothelium over the length of the cell and being dragged along the vascular surface in the direction of blood flow (86). The final stage of vascular interaction prior to transmigration is stationary adhesion where the *B. burgdorferi* cell is at a complete stop and no longer progressing in the direction of the blood stream. Borrelial cells maintained stationary adhesion for an average of 10 min. 3D visualization was performed to determine the localization of interactions in relation to endothelial junctions labeled with PECAM-1 that found stationary adhesion primarily occurred at the junctions while tethering and dragging took place along the endothelial cell surface (86). Non-infectious borrelial cells lacking genes for OM lipoproteins did not display the same interactions with the endothelium as infectious *B. burgdorferi* indicating pathogen ligands that can bind to specific host receptors (87). An *in vitro* flow chamber model was developed to evaluate *B. burgdorferi* interactions with human endothelia under a controlled environment for shear force (89). *B. burgdorferi* endothelial interactions in postcapillary venules by IVM and those in the flow chamber were consistent, thus providing an opportunity to understand the specific mechanics behind tethering, dragging, and stationary adhesion in controlled conditions.

Numerous borrelial lipoproteins have previously been shown to bind components of the ECM (39, 41, 90–94). Norman et al. examined potential mechanisms mediating the interactions with the vasculature through fibronectin (Fn) and glycosaminoglycans (GAGs) (87). Biofluorescent *B. burgdorferi* was incubated with dextran sulfate to block potential GAG interactions and evaluated *in vivo* by epifluorescence IVM resulting in a 30 and 80% reduction in transient and dragging interactions, respectively. Stationary adhesion was reduced to a similar level as dragging when cells were treated with dextran sulfate (87). This indicates that GAGs are involved in mediating the host–pathogen interaction of *B. burgdorferi* in the vasculature. The role of Fn in *B. burgdorferi* binding to the vasculature was assessed by treating *B. burgdorferi* that expresses fibronectin-binding protein, BBK32, with anti-Fn IgG prior to IV injection. Short-lived reduction in transient and dragging interactions were observed, suggesting that Fn plays an important role in the ability of *B. burgdorferi* to interact with the host vascular endothelium (87). Fibronectin C-terminal heparin-binding domain and integrin-interacting cell-binding domain that were responsible for *B. burgdorferi* vascular interaction was evaluated by competition with peptides representing the binding domains under *in vivo* conditions. *B. burgdorferi* was able to readily interact with the endothelium at all stages when the RGD sequence of the integrin-interacting cell-binding domain was in circulation to compete with plasma Fn for binding. Heparin-binding domain peptide decreased *B. burgdorferi* transient interactions by 52% and further reduced dragging and stationary adhesion by 84%, suggesting that host GAGs are responsible the observed vascular interactions. The heparin-binding domain of Fn and GAGs are directly or indirectly important for *B. burgdorferi* interactions with the host endothelial surface.

BBK32 is one of several *B. burgdorferi* proteins shown to bind Fn and its role in mammalian infectivity has been assessed as a ligand under *in vivo* conditions (83, 87, 88, 95–97). Fibronectin binding by *B. burgdorferi* may build a bridge for binding integrins allowing further host–pathogen interactions to occur (92, 98, 99). *B. burgdorferi* lacking *bbk32* attenuates the infectivity phenotype and increases the ID_50_ about 15-fold as determined by cultivation of murine tissues 3 weeks after inoculation (95). High dose infection, specifically 10^5^, with the *bbk32* mutant strain results in colonization of disseminated tissues at a slightly lower level than wild-type when bacterial loads are quantitatively determined (97). *In vivo* imaging of bioluminescent *bbk32* deletion strain demonstrated a similar dose dependent phenotype, but also that the role for *bbk32* may be more important for earlier stages of infection (83). Light emission from mice infected with this strain was significantly less 1 week after inoculation when compared to wild-type infected controls. Gain-of-function experiments utilized non-infectious *B. burgdorferi* lacking several plasmids and an adhesin protein necessary for dissemination or invasion (86–88). Non-infectious *B. burgdorferi* that was complemented with *bbk32* driven by the *ospC* promoter caused increased vascular interactions relative to the non-infectious parent strain lacking the transgene (87). Mutagenesis of *bbk32* in infectious *B. burgdorferi* allowed the specific role of this fibronectin-binding protein to be characterized for Fn binding and vascular interactions (88) (Figure 2). Biofluorescent *bbk32* deletion strain displayed a 20 and 50% reduction in the murine skin flank and joint, respectively, in tethering and dragging. Epifluorescence IVM requires a high dose inoculation by IV injection and may contribute to the different outcomes of *bbk32* infectivity studies using lower dose intradermal inoculum that resulted in more pronounced virulence attenuation. The disruption of two regions of *bbk32* (Fn-binding, Δ158–182, or GAG-binding sequences, Δ45–68) caused a reduction of tethering and dragging, respectively, indicating these binding domains serve distinct mechanistic functions for *B. burgdorferi* endothelial interactions. BBK32 has a fast binding association and disassociation with Fn, but GAG binding is longer lasting and more stable (88). These differences in binding kinetics may explain the distinct binding interactions observed by *in vivo* IVM. Flow chamber studies evaluated the specific mechanism by which BBK32 associates with the endothelium and determined it contributes to stabilization of interactions and increases the range of force and shear stress through catch bond mechanism (89). A catch bond occurs when the higher the tensile force the longer the life of a non-covalent bond between a receptor and ligand (100). It is not clear if BBK32 is directly binding the endothelium utilizing lectin-binding sites or if the interactions are occurring through host ligands. Other possible fibronectin-binding proteins, RevA, RevB, and BB0347, were not able to restore vascular interactions in non-infectious *B. burgdorferi* (88). It is likely that a yet to be identified borrelial proteins that are able to bind fibronectin contribute to vascular interactions in addition to BBK32.

It is likely that many more players are involved in vascular interactions and possibly do so in a tissue specific or spatial manner. A prime candidate is borrelial lipoprotein decorin-binding protein A (DbpA) that is able to bind decorin, heparin, and dermatan sulfate GAG (101–103). *dbpA* is co-transcribed with highly conserved *dbpB* that has a decreased decorin-binding ability and does not impact infection to the degree observed for *dbpA* (13, 14, 101, 104–106). Environmental signals, such as temperature, pH, and CO_2_, regulate *dbpBA* expression in a RpoS-dependent manner (107–116). Incubation of *B. burgdorferi* with neuroglial or human umbilical vein endothelial cell (HUVEC) cells induced the expression of *dbpBA* among other virulence associated lipoproteins (117). *B. burgdorferi* lacking *dbpBA* has a severely attenuated infectivity phenotype with reduced colonization of the skin, heart, and joint when needle inoculated, but tick transmission of the *dbpBA* mutant resulted in an infectivity phenotype similar to wild-type (83, 118–122). Over time high dose inoculum of the *dbpBA* mutant can overcome the limitations to dissemination and reach wild-type bacterial burden in tissues despite reduced pathology in the heart and joint (118). Expression of *dbpA* is detectable up to 8 weeks after inoculation in several tissues, which is dissimilar to another RpoS-regulated gene *ospC* that is downregulated about a week after infection (123, 124). The importance of decorin binding by *B. burgdorferi* is further demonstrated in the resistance of decorin-deficient mice to infection (125). Transcripts of *dbpA* are readily detectable from blood during infection, but there is also some indication that DbpA contributes to lymphatic dissemination of *B. burgdorferi* (118). The *dbpBA* mutant has reduced colonization of lymph nodes of immunocompetent mice relative to wild-type *B. burgdorferi*, but there was no difference observed in immunodeficient mice (118). The ability of DbpBA to aid in vascular interactions was assessed using an *in vitro* flow chamber seeded with HUVECs to mimic shear stress of the blood stream (126). Non-infectious *B. burgdorferi* B313 missing numerous plasmids was complemented with *dbpBA* on a shuttle vector and formed the short-lived tethering interaction (126) (Figure 2). DbpA has not been evaluated under *in vivo* conditions with IVM. In addition, its specific contribution to invasion as assessed by its ability to cross cell culture monolayers or to move in *in vivo* transmigration assays has not been determined. Vascular interaction is merely one step in the borrelial pathogenic process with stage each building upon the other for the end result of persistent infection in several mammalian tissues.

## Invasion

Borrelial dissemination and subsequent invasion of mammalian tissues in the latter stages of disease is an essential step for pathogenesis and immune evasion. Invasion of *B. burgdorferi* by entering into a tissue through transmigration or extravasation results in the colonization of secondary infection sites that become the foci of disease. *B. burgdorferi* has the ability to preferentially escape into heart or joint tissue and cross the blood–brain barrier (BBB) stimulating an inflammatory immune response to cause carditis, arthritis, and neuroborreliosis, respectively (1). The endothelial surface of the BBB has an unique organization and increased level of protection in comparison to other endothelial tissues targeted for invasion by *B. burgdorferi*. Invasion of the BBB by *B. burgdorferi* results in activation of the host plasminogen activation system (PAS), matrix metalloproteases (MMP), and host calcium signaling (127–129). It is likely that *B. burgdorferi* utilizes unique invasion strategies in a tissue-specific manner in addition to mechanisms common to other pathogens for entry into immunoprotected niches.

Early host–pathogen studies evaluated the ability of *B. burgdorferi* to cross cell culture monolayers of various types (130–135). Infectious *B. burgdorferi* is able to adhere and penetrate through tight junctions from the apical surface to traverse the monolayer. This interaction with the host and subsequent transmigration is not without consequence as it can result in the enhanced response of neutrophils and T lymphocytes in HUVEC culture (136, 137). Evidence limited to *in vitro* studies indicate that *B. burgdorferi* has the ability to undergo intracellular invasion of human fibroblast, umbilical vein endothelial, synovial, neuronal, and glial cells without the loss of viability (49, 133, 138, 139). These findings lead to the speculation that borrelial cellular invasion is a mechanism for immune evasion and disease modulation, but the significance is unknown, as this has not been observed using an *in vivo* model system. Furthermore, an important effect of *B. burgdorferi* interacting with the host is the alteration of virulence determinants, including Dbp, OspA, and BBA64, which are important for mammalian infection (117, 140). The host–pathogen interactions and the resulting responses of *B. burgdorferi* and host tissue-specific pathways are important for the advancement of the spirochete into secondary colonization sites. The targeting of immunoprotective niches requires non-specific and specific vascular interactions, as described above, followed by transmigration that is likely mediated by several distinct borrelial adhesins.

### *B. burgdorferi*-Elicited Transmigration in the Mammalian Host: The Role of P66

P66 (*bb0603*) is encoded on the borrelial chromosome and forms an OM β-barrel porin with adhesive capabilities to host integrins that contribute to mammalian infection (141–148). *B. burgdorferi* lacking P66 is able to persist in ticks through molting, but is unable to establish infection in the mammalian dermis and is cleared at the site of inoculation within 48 h following inoculation (149, 150). Expression of *p66* is not observed in the tick vector until exposure to a blood meal and continues well into mammalian infection providing further support for the importance of P66 during mammalian infection. P66 engages the innate and adaptive immune response in the murine host as it is required for infection of mice deficient in the innate immune signaling molecules MyD88 and TLR-4 and elicits a specific antibody response against *p66* expressing *B. burgdorferi*. A short-term infection model with intravenously infected Balb/c mice was used to evaluate the tissue localization of wild-type or *p66* mutant *B. burgdorferi* 1 h after inoculation and resulted in a P66-dependent tissue tropism for the ear and heart (94). Disseminated *B. burgdorferi* requires specific adhesins dependent upon the tissue for colonization of distal sites.

The requirement for infectivity could be due to one or both of P66 identified functions: porin and/or integrin-binding activity. P66 was initially identified as an integral membrane porin in *B. burgdorferi* and was further shown to possess channel conductance (141, 142). Recent work by Kenedy et al. characterized the β-barrel structure of P66 and found that it associates with lipoproteins OspA and OspB (143). The second identified function of P66 is its interaction with β_1_ chain and β_3_ chain integrins that are homodimeric proteins with the function of host cellular signaling and interactions with host cell matrices (147, 149, 151, 152). Traditional mutational analysis and a phage-display screen verified β_3_ intergrin binding by P66 (147, 153). The specific binding region for integrin interaction was localized to amino acids 203–209 of P66 (151). Transcriptional analysis of human embryonic kidney cells and endothelial cells exposed to *B. burgdorferi* with or without *p66* indicated significant changes among host genes involved in several pathways including cellular interactions and actin arrangement indicating a potential role in pathogen invasion (152). Interaction of P66 with the host causing potential cellular arrangements may support the transmigration of the pathogen through tight junctions or possibly the intracellular invasion of *B. burgdorferi*. An *in vitro* gentamicin protection study demonstrated *B. burgdorferi* utilizes β_1_ integrin to invade endothelial or fibroblast cells requiring the rearrangement of actin filaments and Src kinase activity (49). Internalized *B. burgdorferi* displayed the round body morphology previously observed when the pathogen is under stress conditions, but the change in shape could also be due to spatial limitations within the host cell. At this time, the physiologic relevance of intracellular *B. burgdorferi* is unclear because evidence that this event also occurs during a natural infection has not been reported.

The requirement for borrelial P66 binding of host β_3_ integrin for infection in the murine experimental model was assessed by Ristow et al. (154). Mice expressing or devoid of β_3_ integrin had the same ID_50_ as wild-type *B. burgdorferi* and were also not infected by the *p66* mutant strain, indicating that β_3_ integrin is not essential for infection and redundant integrin binding by borrelial cells may also support tissue invasion. Two *p66* integrin-binding targeted mutant strains were generated consisting of a regional deletion of amino acids 202 to 208 (BDel202-208) and double point mutation (BD205A,D207A). Phenotypes of the *p66* targeted mutant strains were characterized for infectivity, *in vitro* integrin binding, porin activity, and penetration of endothelial monolayers. BDel202-208 had an attenuated infectivity phenotype with significant reduced bacterial loads in the heart and tibiotarsal joint, while BD205A,D207A decreased in the heart relative to wild-type *B. burgdorferi* when mice were subcutaneously infected. Intravenous infection slightly alters the kinetics of borrelial infection and only BDel202-208 had a reduced bacterial burden in the heart. BDel202-208 and BD205A,D207A significantly reduced α_V_β_3_ integrin binding relative to wild-type, but did not disrupt channel conductance and maintained porin activity (147, 154). In correspondence with integrin binding, BDel202-208 and BD205A-D207A had an impaired ability to cross endothelial monolayer when compared to the wild-type strain. It is well established that P66 and, more specifically, the region encompassing the integrin-binding region is important for *B. burgdorferi* infection and possibly invasion. The route of host interaction is possibly through β_1_ integrin in addition to β_3_ integrin. β_1_ integrin could not be directly evaluated *in vivo* due to lethality associated with β_1_ deficiency.

The potential that P66 was involved in the dissemination or invasion of *B. burgdorferi* was evaluated by *in vivo* IVM for vascular interactions and in a newly developed high resolution intravital transmigration assay (155). Previous studies utilizing *in vivo* IVM focused on vascular interactions in the skin (86–88). *Cd1d^−/−^* mice are a preferred strain to examine *B. burgdorferi* infection in the joint because the lack of invariant natural killer T cells (iNKT) increases the bacterial burden and the likelihood of visualizing spirochetal vascular interactions or transmigration (156, 157). Kumar et al. focused on the vascular interactions in joint proximal tissue of *Cd1d^−/−^* mice that were capable of recapitulating tethering, dragging, and stationary adhesion interactions of wild-type *B. burgdorferi* observed in murine skin (155). Spinning disk laser confocal microscopy captured a back and forth motility of *B. burgdorferi* as it transmigrates in the joint one day after high dose intravenous inoculation of *Cd1d^−/−^* mice (155). *B. burgdorferi* lacking *p66* showed no difference in tethering, dragging, or stationary adhesion relative to wild-type. Therefore, this adhesive porin is not involved in vasculature interactions. Transmigration and clearance from the bloodstream of *p66* mutants was dramatically reduced when compared to wild-type or complemented *p66* mutant strains raising the question whether the inability of *B. burgdorferi* to invade was due to clearance by the host immune response rather than the ability to cross the epithelial layer. Targeted *p66* mutant strains were also not able to transmigrate, but interestingly, bloodstream clearance is at a similar rate to wild-type indicating that P66 serves an active role in a borrelial transmigration mechanism. *In vivo* localization of β_3_ integrin in the vasculature in relation to *B. burgdorferi* was determined that P66-β_3_ integrin interaction associates with transmigration events at cellular junctions where stationary adhesion most often occurs. *B. burgdorferi* localized to areas in the vasculature with higher concentrations of β_3_ integrin indicating this colocalization is taking place and contributing to transmigration. Studies with β_3_ integrin-deficient mice showed *B. burgdorferi* was able to disseminate to various tissues, suggesting that β_3_ integrin binding is not the sole mechanism for borrelial invasion. It is likely that the pathogen utilizes redundant invasion mechanisms through the binding of other integrins, such as β_1_, or host receptors. Active transmigration in the joint occurs above background levels 24 h after inoculation, which may allow the time for activation of cell signaling pathways in endothelial cells and borrelial adaptation to support transmigration for successful invasion (158). The contribution of BBK32 to endothelial invasion, in addition to the previously noted vascular interactions, is determined not to be involved as the *bbk32* mutant and wild-type *B. burgdorferi* displayed similar rates of transmigration. The role of P66 porin activity in vascular interactions, transmigration, and survival following epithelial invasion is unknown at this time. Taken together, P66 is involved in a potential invade-to-evade mechanism in *B. burgdorferi* that may have additional pathogenic functions (154, 155). Further studies are needed to elucidate possible P66 initiated actin and ECM rearrangements and the impact on the inflammatory response characteristic of Lyme disease.

### *B. burgdorferi* Protease HtrA Role in Invasion

The high-temperature requirement (HtrA) family of ATP-independent serine proteases serve as chaperones, support membrane integrity by degrading damaged or improperly folded proteins, and pathogenesis by processing virulence determinants or degrading components of the ECM in other bacterial pathogens (159, 160). *B. burgdorferi* chromosomally encodes a single HtrA protease (BbHtrA) at *bb0104* (13, 14). BbHtrA exhibits proteolytic activity and the potential of this protein to support borrelial pathogenesis and physiology was the subject of a MicroCommentary in Molecular Microbiology and a perspectives article in Frontiers in Cellular and Infection Microbiology (161–165). BbHtrA is a surface exposed protein that localizes to both the soluble and membrane fractions and can also form oligomeric structures (163, 164, 166). Production levels of BbHtrA are higher at 37°C relative to 34°C correlating with higher activity levels and induction during the stationary phase of *in vitro* cultivation conditions (167). It was the first HtrA in which proteolytic activity inhibited by zinc was described (166). Cell fission protein BB0323 is required for mammalian infection and proteolytically cleaved by BbHtrA demonstrating that *B. burgdorferi* is able to modify its own proteins (168). The *B. burgdorferi htrA* mutant strain does not contain processed BB0323 at elevated temperatures validating the proteolytic function of BbHtrA *in vivo* (167). BbHtrA targets proteins for proteolysis that vary in function including cell fission (BB0323), chemotaxis (CheX), laminin binding (BmpD), and integrin binding (P66) (163, 168, 169). Additional borrelial proteins are bound by BbHtrA without undergoing degradation, indicating that BbHtrA may also function as a chaperone (163). Transcripts of *p66* in a borrelial strain overexpressing *BbhtrA* were reduced demonstrating another layer of regulation employed by this borrelial protease (169). The impact of BbHtrA on pathogenesis is widespread in regards its diverse mechanisms of regulation.

**Figure 3:**
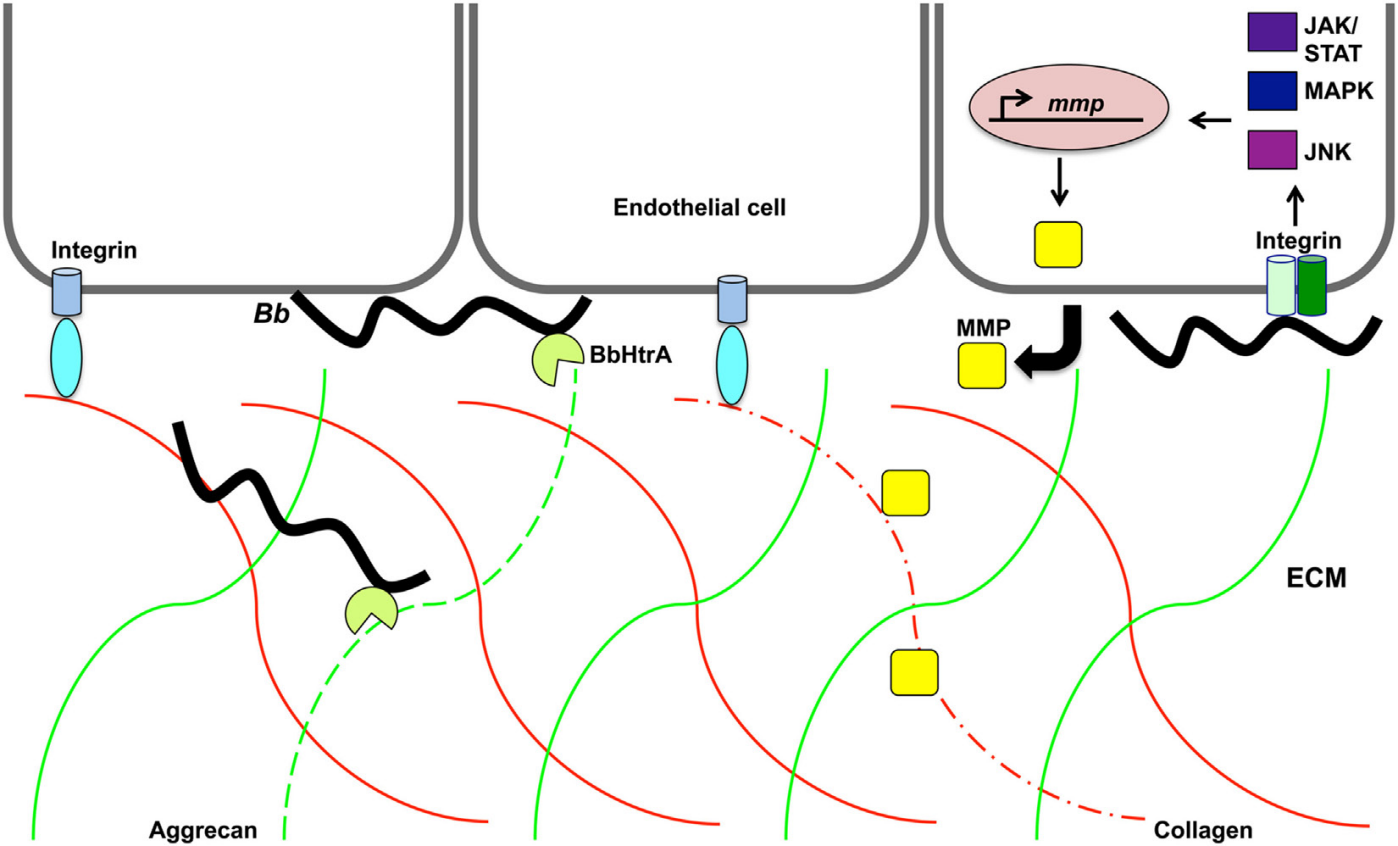
**Proteolytic activity may promote *Borrelia burgdorferi* invasion of the extracellular matrix**. *B. burgdorferi* (*Bb*) protease BbHtrA cleaves the family of hyalectin proteoglycans that includes hyalectin family of chondroitin sulfate proteoglycans, including aggrecan, brevican, neurocan, and versican. Specifically, BbHtrA protease activity degrades aggrecan (green lines) as indicated by dotted green line. This activity may contribute to ECM degradation and allow invasion of the pathogen. The presence of *B. burgdorferi* induces host matrix metalloproteases (MMP) from endothelial cells, chondrocytes, neutrophils, lymphoblast, and keratinocytes. Collagen (red lines) is degraded (dotted red line) by MMP-1 that is transcriptionally regulated along with MMP-3 through the JNK, mitogen-activated protein kinase, and JAK/STAT pathways.

Another host target of borrelial proteolysis is aggrecan, which is a large aggregating proteoglycan found in cartilage with multiple functional domains consisting of two GAG-attachment domains and three globular domains (170). Behera et al. found that *B. burgdorferi-*induced aggrecanase 1 and 2 (ADAMITS 4 and 5) in human chrondrocyte cells causes the cleavage of aggrecan, thus possibly explaining cartilage damage associated with Lyme disease (171). The binding ability of *B. burgdorferi* aggrecan was attributed to borrelial GAG-binding protein (Bgp) (encoded by *bb0588*) and BbHtrA (164). The aggrecan interglobular domain (IGD), which resides between globular domains 1 and 2 (G1 and G2), is specifically cleaved by BbHtrA, thereby disrupting its ability to associate with GAG (164, 170). Proteolytic assays were performed to identify other host ECM proteins degraded by BbHtrA (165). The hyalectin family of chondroitin sulfate proteoglycans, including aggrecan, brevican, neurocan, and versican, were also degraded by BbHtrA (Figure 3). Additional BbHtrA degraded ECM proteins were identified as fibronectin, biglycan, and decorin. *B. burgdorferi* is known to encode specific adhesins with the ability to bind these proteoglycans. Cell junction protein E-cadherin was only moderately degraded while collagen II and tenascin C were unaffected by incubation with recombinant BbHtrA. Together, these findings indicate that BbHtrA also has target specificity for degradation of host ECM. A point mutation was generated in the active site of BbHtrA, designated BbHtrA^S226A^, eliminating the protease activity and resulting in the loss of degradation of host ECM proteins (164, 165). The potential physiologic significance of BbHtrA proteolytic and chaperone activity was confirmed when Ye et al. who generated an *htrA* deletion mutant strain that was not recoverable by cultivation from murine tissues two weeks following inoculation (167). It cannot be determined if the loss of infectivity was due to lack of proper borrelial protein processing through degradation and chaperone activity, a reduction in host ECM degradation that may inhibit invasion, or a compounding effect of both functions. The absence of *htrA* results in *B. burgdorferi* cells with *in vitro* growth deficiencies, cell membrane blebbing, and clustering of cells at elevated temperature when BbHtrA has the highest level of activity (167). These described *in vitro* phenotypes of *B. burgdorferi* would significantly impair infection if also observed *in vivo*. BbHtrA is highly immunogenic causing the stimulation of inflammatory signaling and possibly causes further damage of the host ECM (164, 165). The immunogenicity of BbHtrA did not translate into protection against infection in the murine model (172). *B. burgdorferi* may utilize the multifunctional BbHtrA to promote invasion through its ability to regulate and/or process virulence determinants along with the degradation of the host ECM.

#### Induction of Host Proteases in Response to *B. burgdorferi*

*Borrelia burgdorferi* induction of aggrecanase activity was first observed in the synovial fluid of patients with Lyme arthritis and was thought to possibly be caused by host MMPs (173). MMPs are secreted zinc and calcium-dependent host proteases that act to remodel the ECM by specific degradation of aggrecan, collagen, elastin, fibronectin, and gelatin (174). MMPs aid in tissue growth, repair, and remodeling of healthy tissues. These host proteases also cleave chemokines, cytokines, and the accompanying receptors causing an inactivation or activation of these immunological molecules. The degraded ECM fragments released stimulate a localized inflammatory response similar to that observed in Lyme disease manifestations. *B. burgdorferi* induces a range of MMPs that are dependent upon the types of cell stimulated and infecting borrelial strain (173, 175–177). Human neutrophils, lymphoblast, and keratinocytes, secrete pro-MMP-9 when exposed to *B. burgdorferi* (175). HUVECs were able to release pro-MMP-9 and pro-MMP-2 under the same conditions. Borrelial infection induces the production of collagenase-1 (MMP-1), gelatinase-2 (MMP-2), stromelysin-1 (MMP-3), and gelatinase B (MMP-9) in synovial fluid from patients with erythema migrans in addition to aggrecanase activation (173, 178) (Figure 3). The aggrecanase activity observed in synovial fluid of Lyme disease patients was found not to be associated with the stimulated MMP-2, MMP-3, or MMP-9, but may have been due to the more recently characterized BbHtrA activity described above (173). Cartilage explants from rhesus monkey and bovine were used as an *in vitro* model of Lyme arthritis and incubation with *B. burgdorferi* resulted in cartilage degradation that was prevented in the presence of MMP inhibitors (173). This could provide an explanation for the damage observed with Lyme arthritis and shed light on mechanisms utilized by *B. burgdorferi* for invasion and modulation of disease. The *B. burgdorferi-*induced profile of MMPs is specific to the tissue type and host species (173, 175). It was shown that both similarities and differences exist in the types of MMPs induced by *B. burgdorferi* in mice and humans and could be the reason for differences in susceptibility to borrelial infection and the development of arthritis. Different MMPs activated in response to *B. burgdorferi* are regulated through distinct host pathways (179–181). Specifically, MMP-1 and MMP-3 are regulated through c-Jun N-terminal kinase (JNK), p38 mitogen-activated protein kinase (MAPK), and Janus kinase/signal transducer and activation of transcription (JAK/STAT) pathway (179) (Figure 3). MMP-1, MMP-3, and MMP-9 regulation occurs through toll-like receptor 2 (TLR-2) (180, 181). MMP-1 and MMP-3 are also induced through the interaction of borrelial cells with α_3_ integrins (181). *B. burgdorferi* may utilize MMPs to manipulate ECM remodeling to promote invasion of the pathogen for secondary colonization of tissues with the diversity of induced MMPs and regulatory pathways ensuring progression of disease.

The idea that MMPs could promote invasion was supported by the increased transmigration of *B. burgdorferi* through ECM components under *in vitro* conditions when incubated with MMP-9 (175). MMP-9 is able to cleave collagen type I and the resulting fragments chemoattract peripheral blood mononuclear cells; thus, this activity also elicits an immune response that could limit borrelial pathogenesis (182). The specific role of MMP-9 in *B. burgdorferi* invasion and inflammatory response under *in vivo* conditions is assessed by infecting MMP-9^−/−^ mice and comparing to wild-type mice in regards to bacterial burden, arthritis, and carditis. Murine hearts and joints were colonized at similar levels of bacterial burden indicating that MMP-9 alone is not responsible for invasion. This observation does not completely rule out the involvement of MMP-9 in aiding *B. burgdorferi* invasion of tissues through ECM remodeling. It is likely redundant mechanisms that are utilized to guarantee successful pathogenesis, which may occur through other MMPs or BbHtrA. Infected MMP-9^−/−^ mice develop less arthritis relative to wild-type mice with an unexpected similarity in cytokine and chemokine expression, indicating that the ability of MMP-9 to modulate an inflammatory response is independent of *B. burgdorferi* infection. Further investigation of other MMPs individually or in combination is needed to fully understand the contribution of MMPs to *B. burgdorferi* invasion.

Plasmin has been observed in multiple studies to be activated by *B. burgdorferi* and is able to degrade ECM components and activate MMPs (173, 175, 183). The PAS, also referred to as the fibrinolytic system, involves the induction of a series of proteases and accompanying regulatory inhibitors for the purpose of fibrin cleavage that support tissue remodeling, healing, and cell migration (129, 184). It was suspected that the activation of plasmin by the presence of *B. burgdorferi* could serve as a potential mechanism for spirochetal invasion. *B. burgdorferi* readily binds plasminogen and is converted to plasmin by host-derived urokinase plasminogen activator (uPA) (38, 185, 186). This tightly regulated process is kept in check by plasminogen inhibitors type 1 and 2 (PAI-1 and PAI-2). Plasminogen binds *B. burgdorferi* through numerous identified receptors and promotes the transmigration of the pathogen across HUVEC monolayers (187). MMP-9 induction was observed in the presence of plasminogen when human PMBC cells were co-incubated with *B. burgdorferi* (175). During a tick blood meal, plasminogen is activated, but is not required for borrelial transmission to a murine host (188). uPA is induced in monocytes of mice and humans in the presence of *B. burgdorferi* (189, 190). Hovius et al. demonstrated that borrelia induces the PAS and, more specifically, showed that the receptor of uPA controls bacterial burden and promotes the phagocytosis of *B. burgdorferi* by leukocytes (191). uPAR-deficient mice had increased *B. burgdorferi* colonization relative to the C56BL/6 wild-type mice. However, this increase was not observed in uPA, tPA, or PAI-1 knockout mice. *In vitro* phagocytosis assays display limited leukocyte phagocytosis in the absence of uPAR, but not the other members of the PAS system. Therefore, the host PAS is not hijacked by *B. burgdorferi* for ECM remodeling leading to the conclusion that this system does not contribute to invasion. However, this conclusion does not rule out the contributions of tick salivary glands or other supporting pathogenic mechanisms in mediating transmigration.

## Summary

The success of *B. burgdorferi* as a pathogen involves dynamic adaptation and interactions of specific tissues of the mammalian host with the pathogen during each stage of disease. *B. burgdorferi* engages numerous adhesins that are important for mammalian infection, but the specific contributions of these adhesins for dissemination or invasion are not fully elucidated. The specific mechanisms known to contribute to borrelial dissemination in the form of vascular interaction and invasion by transmigration under *in vitro* conditions or during mammalian infection has been the focus of this review. It is clear that *B. burgdorferi* is a resilient bacterium as it relies on redundant functions for both dissemination and invasion to ensure progression of pathogenesis. This presents a challenge to clearly understand these mechanisms. Significant advances in our understanding of *B. burgdorferi* vascular interactions and invasion have been made through the development of *in vivo* imaging technologies allowing the identification of stages of interactions. Borrelial adhesins are responsible for interactions with the host that promote dissemination or invasion in a tissue specific manner. It is unclear the degree to which *B. burgdorferi* interaction with the endothelium can directly induce cellular rearrangement allowing for the invasion of host tissues. Furthermore, pathogen and host proteases combat a barrier to *B. burgdorferi* invasion by proteolytically cleaving the ECM.

## Author Contributions

The only contributor to this manuscript is JH.

## Conflict of Interest Statement

The author declares that the research was conducted in the absence of any commercial or financial relationships that could be construed as a potential conflict of interest.
